# Diabetes Registry of Pakistan

**DOI:** 10.12669/pjms.36.3.1877

**Published:** 2020

**Authors:** Abdul Basit, Asher Fawwad, Kulsoom Baqa

**Affiliations:** 1 Abdul Basit, FRCP. Professor of Medicine (BMU), Director (BIDE), Baqai Institute of Diabetology and Endocrinology (BIDE), Karachi, Pakistan. Baqai Medical University (BMU), Karachi, Pakistan; 2 Asher Fawwad, Chairman and Professor of Biochemistry, Department of Biochemistry (BMU), Research Director (BIDE), Baqai Institute of Diabetology and Endocrinology (BIDE), Karachi, Pakistan. Baqai Medical University (BMU), Karachi, Pakistan; 3 Miss. Kulsoom Baqa, M.Phil. Research Officer, Research Department (BIDE-BMU), Baqai Institute of Diabetology and Endocrinology (BIDE), Karachi, Pakistan. Baqai Medical University (BMU), Karachi, Pakistan

**Keywords:** Registry, Diabetes, Pakistan, National diabetes survey, Incidence

## Abstract

Diabetes registries can be used to monitor the prevalence and incidence of diabetes. Diabetes registries are used in many countries for population management of diabetes, outcomes management, and development of Clinician finding Support Structure, for example, National Diabetes Registry (NDR) in Sweden and Singapore diabetes registry. According to 2nd National Diabetes Survey of Pakistan (NDSP) 2016-2017, overall 26.3% adult (≥20) suffer from diabetes (27.4 million people). Health Research Advisory Board (HRAB) of Pakistan has initiated the mission of developing disease registries countrywide. Diabetes Registry of Pakistan (DROP) under the supervision of Prof. Abdul Basit and Dr. Asher Fawwad to enumerate the degree of the national disease burden. For type 1 diabetes, Diabetes Registry of Pakistan for type 1 (DROP-1) has already started. DROP-1 can be a good pilot arm for developing the robust methodology for DROP. The upcoming and the ongoing research is periodically being linked with the registry to ensure its modernization. This registry is a useful tool for tracking the status of patient, in order to limit the burden of data collection. A web-based data entry system and automated random sampling has enabled useful data collection and tracking with relatively minimal effort.

A registry is an organized system that uses observational study method for collection of uniform data to evaluate specified outcome for a population.^1^ Diabetes registries can be used to monitor the prevalence and incidence of diabetes, to provide a sampling frame for epidemiologic and clinical studies, and provide information to health service providers and planners on risk factors and complications.^2^ Diabetes registries are used in many countries for population management of diabetes, outcomes management, and development of Clinician finding Support Structure, just like, National Diabetes Registry (NDR) in Sweden^3^ and Singapore diabetes registry are being used.^4^ However, data from Pakistan is very limited.

According to an assessment of 2017, 415 million people globally suffering from diabetes and it is projected that the number will surge to 629 million people by 2045.^5^ Around, 70% of the cases are existing in the developing countries. According to 2nd National Diabetes Survey of Pakistan (NDSP) 2016-2017, overall 26.3% adult (≥20) suffer from diabetes (27.4 million people).^6^

A study by Staines et al reported type 1 diabetes incidence rates: 1.0 cases per 100,000 children <17 years of age per year in Karachi between 1989-1993.^7^ These are low compared to rates of 4.2-40.9 in the European populations reported in the same period.^8^ Although, there are clear geographic differences in trends but the estimated annual increase in T1D is around 3%.^9^

Health Research Advisory Board (HRAB) of Pakistan has initiated the mission of developing disease registries countrywide. With the collaboration of HRAB, Baqai Institute of Diabetology and Endocrinology (BIDE) has recently began the Diabetes Registry of Pakistan (DROP) under the supervision of Prof. Abdul Basit and Dr. Asher Fawwad to enumerate the degree of the national disease burden. For type 1 diabetes, Diabetes Registry of Pakistan for type 1 (DROP-1) has already started.^10^ DROP-1 can be a good pilot arm for developing the robust methodology for DROP.^11^

In DROP, the information is collected through an electronic portal with a separate login details assigned to health care professionals from participating institutes. Online data collection Forms are filled. Mandatory fields are required to be filled for a successful entry. Paper questionnaires are used in remote areas for collecting the data. These questionnaires are then delivered to BIDE for manual entry into the electronic portal.

There are a number of challenges in the process of having successful DROP. Main hurdle is lack of awareness among health care providers regarding the significance of health registries in the country including DROP. Moreover, the health infrastructure of Pakistan is yet not sufficiently developed. Specialized diabetes centers, particularly in rural and peripheral urban areas are only very few in numbers. Minimal health budget hardly is enough for disease management, lest supporting registries. High patients – doctor ratio further limits time for data collection and updating registries. Low literacy rate and generalized community unawareness aggravates the situation and departments health does not find disease registries on their top priorities.

To overcome these challenges, many measures have been taken. Communication and interaction with the government and health authorities is being established in order to sensitize them on the significance of DROP. Within the existing health infrastructure comprising of the public and the private sector, the benefits and implications of DROP are being demonstrated to the health care providers and they are being involved in the process. Although there is limited number of specialized diabetes centers in the country, a network of primary care physicians trained in the diabetes care (Diploma in Diabetology) exists across the country. These primary care diabetes physicians are being involved in the process of DROP. Measures have been taken to minimize the cost and expenses of the registry and keep these within the allocated funding and budget. More user-friendly data collection tools have been provided and multiple incentives are being offered to the patients and doctors. The efficient surveillance and monitoring of the registry have been generated. Regular coordination and communication have been established with the respective stakeholders. Periodic reporting of data to higher authorities is being implemented.

These measures would be helpful in linking the registry with legislation and policy making institutions. On the other hand, the upcoming and the ongoing research is periodically being linked with the registry to ensure its modernization.

This registry is a useful tool for tracking the status of patient, in order to limit the burden of data collection. A web-based data entry system and automated random sampling has enabled useful data collection and tracking with relatively minimal effort.

## Authors’ Contributions:

**AB and AF:** Concept, design, interpretation of data, edited and approved the manuscript.

**KB:** Literature search, interpretation of data and prepared the manuscript.

## References

[1] Gliklich RE, Dreyer NA, Leavy MB (2014). Registries for evaluating patient outcomes:a user's guide, Rockville. MD:Agency for Healthcare Research and Quality.

[2] Lakshminarayanan S, Kar SS, Gupta R, Xavier D, Reddy SV (2017). Primary healthcare-based diabetes registry in Puducherry:Design and methods. Indian J Endocrinol Metab.

[3] Gudbjörnsdottir S, Cederholm J, Nilsson PM, Eliasson B (2003). Steering Committee of the Swedish National Diabetes Register. The National Diabetes Register in Sweden:An implementation of the St. Vincent Declaration for quality improvement in diabetes care. Diabetes Care.

[4] Toh MP, Leong HS, Lim BK (2009). Development of a diabetes registry to improve quality of care in the National Healthcare Group in Singapore. Ann Acad Med Singapore.

[5] Cho NH, Shaw JE, Karuranga S, Huang Y, da Rocha Fern, andes JD, Ohlrogge AW (2018). IDF Diabetes Atlas:global estimates of diabetes prevalence for 2017 and projections for 2045. Diabetes Res Clin Pract.

[6] Basit A, Fawwad A, Qureshi H, Shera AS (2018). Prevalence of diabetes, pre-diabetes and associated risk factors:Second National Diabetes Survey of Pakistan (NDSP) 2016–2017. BMJ Open.

[7] Staines A, Hanif S, Ahmed S, McKinney PA, Shera S, Bodansky HJ (1997). Incidence of insulin dependent diabetes mellitus in Karachi, Pakistan. Arch Dis Childhood.

[8] DIAMOND Project Group. ncidence and trends of childhood Type 1 diabetes worldwide 1990–1999 (2006). Diabetic Med.

[9] Ahmedani MY, Fawwad A, Shaheen F, Tahir B, Waris N, Basit A (2019). Optimized health care for subjects with type 1 diabetes in a resource constraint society:A three-year follow-up study from Pakistan. World J Diabetes.

[10] Fawwad A, Ahmedani MY, Basit A (2017). Integrated and comprehensive care of people with type 1 diabetes in a resource poor environment- 'insulin my life (IML)”project. Int J Adv Res.

[11] Basit A, Fawwad A, Baqa K (2019). Pakistan and diabetes—A country on the edge. Diabetes Res Clin Pract.

